# CD20 expression sub-stratifies standard-risk patients with B cell precursor acute lymphoblastic leukemia

**DOI:** 10.18632/oncotarget.22207

**Published:** 2017-10-31

**Authors:** ShenMiao Yang, Jing Wang, Ting Zhao, JinSong Jia, HongHu Zhu, Hao Jiang, Jin Lu, Bin Jiang, HongXia Shi, YanRong Liu, YueYun Lai, LanPing Xu, XiaoJun Huang, Qian Jiang

**Affiliations:** ^1^ Peking University People's Hospital, Peking University Institute of Hematology, Beijing, China; ^2^ Peking Tsinghua Center for Life Sciences, Academy for Advanced Interdisciplinary Studies, Peking University, Beijing, China

**Keywords:** CD20, adult, Ph-negative, B cell precursor acute lymphoblastic leukemia

## Abstract

Patients with standard-risk adult acute lymphoblastic leukemia (ALL) treated with chemotherapy do not have satisfactory outcomes. To more precisely classify ALL patients and optimize treatment, we re-evaluated the risk stratification system by examining CD20 expression and other classic risk factors at diagnosis. We retrospectively analyzed response to induction chemotherapy of 217 consecutive patients with newly diagnosed Philadelphia-negative B cell precursor-ALL. Survival analyses were conducted for the 136 patients who were intended to be treated with chemotherapy alone. Among the 217 patients, 69 (31.8%) were considered standard risk based on age <35 years, white blood cell count <30 × 10^9^/L, absence of central nervous system involvement, and high-risk cytogenetic abnormalities. Seventy-four patients (34.1%) expressed CD20 on ≥20% of leukemia blasts and were considered CD20 positive. We found that fewer CD20-positive than CD20-negative patients achieved durable first complete responses (CR1 ≥3 months) (81.1% vs. 94.9%, P=0.002). Within the standard-risk group, more CD20-negative than CD20-positive patients achieved CR (100% vs. 83.3%, P=0.003) and durable CR1 (100% vs. 82.4%, P=0.014). For patients in the CD20-negative standard-risk, CD20-positive standard-risk, CD20-negative high-risk, and CD20-positive high-risk groups, the 3-year cumulative incidence of relapse was 42.6%, 70.0%, 59.3%, and 69.5%, respectively (P=0.118); the 3-year disease-free survival rates were 52.1%, 0%, 20.7%, and 13.7%, respectively (P=0.006); and the 3-year overall survival rates were 55.8%, 13.8%, 23.6%, and 16.9%, respectively (P=0.006). Our results suggest that patients with CD20-negative standard-risk B cell precursor-ALL have favorable prognosis compared with CD20-positive standard-risk or CD20-negative or -positive high-risk patients. CD20-positive standard-risk ALL patients may need other therapeutic modalities bridging to allogeneic hematopoietic stem cell transplantation.

## INTRODUCTION

Patients with acute lymphoblastic leukemia (ALL) are currently treated with risk-adapted therapeutic strategies and molecularly targeted agents. Classic risk factors for adult patients with ALL at diagnosis include older age [1–3], high white blood cell (WBC) count [3, 4], specific cytogenetic abnormalities [5, 6], and central nervous system (CNS) involvement [7]. Response to induction chemotherapy [4] and minimal residual disease monitored during chemotherapies have also been demonstrated to be significant prognostic factors [8, 9]. For high-risk patients, allogeneic hematopoietic stem cell transplantation (allo-HCT) is accepted as a curative option. However, the 10-year overall survival (OS) rate of patients without high-risk characteristics treated with chemotherapy alone is only 38.9% [10]. This unsatisfactory outcome has prompted some investigators to treat standard-risk patients with allo-HCT at first complete response (CR1) [11–14]. Our experience suggests that standard-risk population is heterogeneous, necessitating the identification of patients who could be spared intensive treatment such as allo-HCT. To this end, we evaluated CD20 expression on leukemic cells, which is a known independent adverse prognostic marker for ALL patients [15, 16] together with the classic risk stratification system for patients with Philadelphia-negative B cell precursor (BCP)-ALL.

## RESULTS

### Patient characteristics

Among the 217 patients evaluated, 111 were male and 106 were female. The median age was 34 years with a range of 18–64 years. Sixty-nine patients (31.8%) were at standard risk. Seventy-four patients (34.1%) expressed CD20 on ALL cells and were considered CD20-positive. The proportion of CD20-positive patients was not significantly different between the high-risk (37.8%) and low-risk groups (26.1%, P= 0.089).

CD20 expression was not associated with classic high-risk factors, including age ≥35 years, WBC ≥30 ×10^9^/L at diagnosis, CNS involvement, and cytogenetic risk. Platelet counts were lower in the CD20-positive than in the CD20-negative group (P=0.006). More CD20-positive than CD20-negative patients had extramedullary disease (EMD) (50.6% vs 28.1%, P=0.001), hepatosplenomegaly and lymphadenopathy (41.6% vs 26.3%, P=0.006), and CNS involvement (10.4% vs 1.9%, P=0.006) (Table 1).

**Table 1:** Patient characteristics

	CD20 positive n=74	CD20 negative n=143	P
Sex (M/F)	39/35	72/71	0.742
Age (median, range)	37.0 (18-64)	33.0 (18-61)	0.486
WBC (median, range)	8.72 (1.0-379.37)	8.97 (1.09-563.57)	0.447
HB (median, range)	92.0 (51.0-152.0)	85.5 (38.0-158.0)	0.027^*^
PLT (median, range)	41.5(3.0-285.0)	67(5.0-391.5)	0.014^*^
EMD (n, (%))	39(52.7)	38(26.6)	<0.001^**^
Hepatosplenomegaly and lymphadeopathy (n, (%))	32 (43.2)	35 (24.5)	0.005^**^
CNSL (n, (%))	8 (10.8)	3 (2.1)	0.009^**^
Cytogenetic risk (n, (%))			0.486
Low	1 (1.4)	2 (1.4)	
Intermediate	44 (59.5)	94 (65.7)	
High and very high	29 (39.2)	47(32.9)	
11q23/MLL translocations	0	13	
t(1;19)	2	5	
Complex karyotype	8	9	
-7, del(7)	6	7	
+8	4	6	
low hypodiploidy (30-39)	9	7	
Risk stratification			0.089
Standard risk	18 (24.3)	51 (35.7)	
High risk	56 (75.7)	92 (64.3)	

### Response

Complete response (CR) was achieved with the first course of induction chemotherapy (defined as CRearly) in 176 patients (81.1%), and after one or more courses of chemotherapy (CRfinal) in 205 patients (94.5%). We defined durable CR1 as CR ≥3 months representing a reasonable time period to prepare for allo-HCT. Of the 217 patients, 195 (89.9%) achieved durable CR1.

The CD20-positive and CD20-negative groups did not differ in the proportion of patients achieving CRearly (81.8% vs. 79.7%, respectively; P=0.709) or CRfinal (96.5% vs. 90.5%, respectively; P=0.113). However, fewer CD20-positive than CD20-negative patients achieved durable CR1 (81.1% vs. 94.9%, P=0.002).

When analyzed according to risk status, the standard-risk and high-risk patients did not differ in the proportion achieving CRearly (84.1% vs 79.7%, respectively; P=0.448), CRfinal (95.7% vs. 93.9%, respectively; P=0.756), or durable CR1 (95.5% vs 91.9%, respectively; P=0.123). However, within the standard-risk group, more CD20-negative than CD20-positive patients achieved CRfinal (100% vs. 83.3%, respectively; P=0.003) and durable CR1 (100% vs. 82.4%, respectively; P=0.014). The CD20-negative and CD20-positive standard-risk patients did not differ in the rate of CRearly (88.2% vs. 72.4%, respectively; P=0.140). In the high-risk group, there were no differences in CR rates between CD20-positive and CD20-negative patients (Table 2).

**Table 2:** Patient response according to risk status and CD20 expression

	Standard risk			High risk			P
	CD20-negative	CD20-positive	P	CD20-negative	CD20-positive	P	
durable CR1≥3 months	100%	82.4%	0.014^*^	92.4%	85.2%	0.166	0.123
CR after the first course of chemotherapy (CRearly)	88.2%	72.2%	0.140	78.3%	82.1%	0.569	0.448
CR after any course of induction chemotherapies(CRfinal)	100%	83.3%	0.003^**^	94.6%	92.9%	0.730	0.030^*^

### Long-term outcomes

Of the 217 BCP-ALL patients, 81 patients received allo-HCT in CR1. The outcomes of those patients are not discussed here. The remaining136 were intended to be treated with chemotherapy alone. Within this subset, platelet counts were lower and extramedullary disease (EMD) was more common in the CD20-positive patients than in CD20-negative patients. However, the proportion of patients with hepatosplenomegaly and CNS leukemia (CNSL) was not significantly different between the two groups (Table 3). And the proportion of patients treated with consolidation regimens of hyperCVAD after CR1 was not significantly different. The median follow-up was 33.0 (range 10.0–170.0) months. The 3-year cumulative incidence of relapse (CIR) was 64.4%, of non-relapse mortality was 14.3%, of disease-free survival (DFS) was 21.0%, and of OS was 25.3%.

**Table 3:** Characteristics of patients intended to be treated with chemotherapy

	CD20 positive n=55	CD20 negative n=81	P
Sex (M/F)	27/28	41/40	0.861
Age (median, range)	37.0 (18-64)	35.0 (18-61)	0.401
WBC (median, range)	10.85 (1.58-379.37)	8.66 (1.2-563.57)	0.928
HB (median, range)	92.0 (39.2-152.0)	85.5 (38.0-154.0)	0.193
PLT (median, range)	34.5(6.0-272.0)	58.0(7.0-391.5)	0.029^*^
EMD (n, (%))	31 (56.3)	26 (32.1)	0.005^**^
Hepatosplenomegaly and lymphadeopathy (n, (%))	26(47.3)	25 (30.9)	0.052
CNSL (n, (%))	6 (10.9)	3 (3.7)	0.157
Cytogenetic risk (n, (%))			0.123
Low	1 (1.8)	0 (0)	
Intermediate	42 (76.4)	58 (71.6)	
High and very high	12 (21.8)	23 (28.4)	
Risk stratification			0.765
Standard risk	15(27.3)	24 (29.6)	
High risk	40(72.7)	57 (70.4)	
Consolidation regimen			0.500
HyperCVAD	15(27.3)	18 (22.2)	
Non-hyperCVAD	40(72.7)	63 (77.8)	

**Figure 1:**
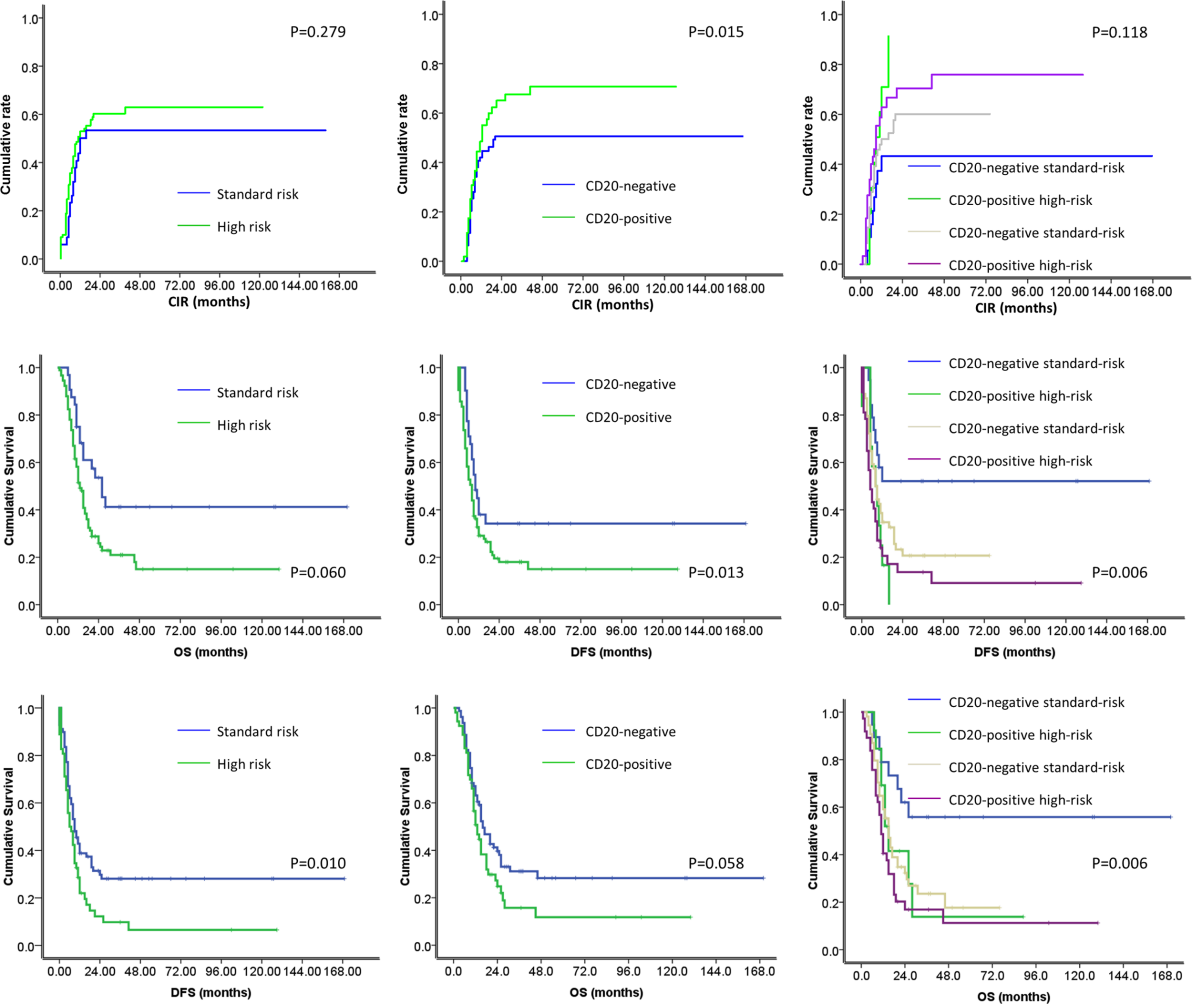
Survival according to risk status and CD20 expression: CD20-positive patients had higher 3-year cumulative incidence of relapse than CD20-negative patients (73. 1% vs. 54.7%, respectively; P=0.015) and lower 3-year disease free survial(12.2% vs. 29.7%, respectively; P=0.013)

We used univariate analysis to evaluate the prognostic significance of the clinical characteristics included in the risk stratifications as well as sex, hemoglobin level, platelet count, CD20 expression, lymphadenopathy, hepatosplenomegaly, and consolidation regimen. High-risk patients had lower 3-year OS than standard-risk patients (25.9% vs. 53.6%, respectively; P=0.007). CD20-positive patients had higher 3-year CIR than CD20-negative patients (73.1% vs. 54.7%, respectively; P=0.015) and lower 3-year DFS (12.2% vs. 29.7%, respectively; P=0.013) (Figure 1). We performed multivariate analysis of factors with a P value <0.20 in the univariate analysis (Table 3). High-risk score was the only independent adverse factor for OS (hazard ratio [HR] 2.055, 95% confidence intervals [CI] 1.199–3.527, P=0.009), and CD20 expression was the only independent adverse factor for DFS (HR 1.917, 95% CI 1.237–2.971, P=0.004) and CIR (HR 1.802, 95% CI 1.061–3.061, P=0.029) (Table 4).

**Table 4:** Univariate and multivariate analysis of risk factors for cumulative incidence of relapse, disease-free survival, and overall survival in patients treated with chemotherapy

	3-year CIR				3-year DFS				3-year OS			
	Univariate analysis		Multivariate analysis		Univariate analysis		Multivariate analysis		Univariate analysis		Multivariate analysis	
	HR(95%CI)	*P*	HR(95%CI)	*P*	HR(95%CI)	*P*	HR(95%CI)	*P*	HR(95%CI)	*P*	HR(95%CI)	*P*
High risk	1.462 (0.833-2.567)	0.186	1.255 (0.687-2.292)	0.460	1.610 (0.981-2.644)	0.060	1.581 (0.961-2.603)	0.071	2.018 (1.184-3.439)	0.010^*^	2.055 (1.199-3.527)	0.009^**^
Factors not included in risk stratification												
Female	1.105 (0.564-2.165)	0.612			0.935 (0.632-1.382)	0.735			0.733 (0.488-1.101)	0.135	0.674 (0.437-1.039)	0.074
HB≥100g/L	0.907 (0.503-1.488)	0.700			0.890 (0.586-1.352)	0.585			0.958 (0.622-1.474)	0.844		
PLT ≥100×10^9^/L	1.962 (1.074-3.586)	0.028^*^	1.773 (0.903-3.482)	0.096	1.668 (1.019-2.730)	0.042^*^	1.308 (0.732-2.337)	0.364	1..516 (0.913-2.518)	0.108	1.173 (0.640-2.149)	0.605
Lymphadeopathy and hepatosplenomegaly	1.120 (0.686-1.827)	0.897			1.180 (0.782-1.779)	0.431			1.264 (0.826-1.934)	0.281		
CD20-positive	1.773 (1.116-2.817)	0.015^*^	1.802 (1.061-3.061)	0.029^*^	1.648 (1.110-2.445)	0.013^*^	1.917 (1.237-2.971)	0.004^*^	1.485 (0.987-2.235)	0.058	1.484 (0.965-2.282)	0.073
Consolidation with hyperCVAD	1.414 (0.889-2.247	0.143	1.574 (0.945-2.622)	0.081	1.308 (0.882-1.939)	0.181	1.463 (0.946-2.261)	0.087	0.991 (0.685-1.492)	0.964		

We next analyzed the outcome of groups combining risk status and CD20 expression. We found that CD20-negative standard-risk patients had a significantly better DFS and OS than did the CD20-positive standard-risk, CD20-positive high-risk, and CD20-negative high-risk patients. Survival did not differ among the latter three groups. The CD20-negative standard-risk, CD20-positive standard-risk, CD20-negative high-risk, and CD20-positive high-risk patients had 3-year CIR rates of 42.6%, 70.0%, 59.3%, and 69.5%, respectively (P=0.118); 3-year DFS rates of 52.1%, 0%, 20.7%, and 13.7%, respectively (P=0.006); and 3-year OS rates of 55.8%, 13.8%, 23.6%, and 16.9%, respectively (P=0.006) (Figure 1).

## DISCUSSION

With this study, we describe that CD20 expression can be helpful in understanding characteristics of standard-risk BCP-ALL patients. CD20-negative patients with standard-risk BCP-ALL had the most favorable outcomes, while the prognosis for CD20-positive standard-risk patients was poor, similar to that of high-risk patients.

Furthermore, CD20 expression was the only independent risk factor for poor DFS in patients treated with conventional chemotherapy alone. This adverse prognostic significance of CD20 expression for survival is consistent with the results of other studies. Thomas et al. reported that the 3-year rates of durable CR and OS were uniformly poor for the CD20-positive group as compared with the CD20-negative group, regardless of the chemotherapy regimen (durable CR 20% vs 55%, P<0.001; OS 27% vs 40%, P=0.03, respectively) [15]. The phase II study of the Group for Research on Adult Acute Lymphoblastic Leukemia (GRAALL), conducted in patients with Philadelphia (Ph)-negative ALL, found CD20 expression to be independently associated with higher CIR (HR=1.9, P=0.045) [16].

CD20 is expressed on both normal and malignant B cells. CD20 is a 33–37 kDa nonglycosylated transmembrane phosphoprotein that forms tetramers and functions in store-operated calcium entry [17]. Studies with small interfering RNA-mediated knockdown of CD20 expression or monoclonal antibody-mediated blocking of CD20 function have shown that this molecule plays an important role in cell-cycle progression and differentiation via downstream signaling pathways. Downregulation of CD20 expression in Ramos cells results in an increase in apoptosis [18]. Rituximab (anti-CD20) treatment preferentially inhibits expression of the antiapoptotic proteins Bcl-2/Bcl-xL via constitutive activation of p38 MAPK, ERK 1/2, NF-κB, and AKT pathways [19]. These molecular studies may explain the pathophysiological and prognostic significance of CD20 expression.

We found that CD20 expression was associated with lower platelet counts and higher rates of EMD, especially CNSL, the latter being a well-accepted poor risk factor. In addition to some of the patient characteristics at diagnosis, their treatment responses were strong indicators of outcome. The low rate of durable CR1 observed in patients with ALL suggests an unsatisfying depth of response, which is also associated with poor outcome.

In previous studies, classic risk factors, such as age ≥35 years, WBC count ≥30 × 10^9^/L, CNSL, and high-risk chromosomal abnormalities, did not seem to affect the response rates of patients with BCP-ALL [15, 16]. This is consistent with our inability to detect a significant difference in the response rates between standard- and high-risk groups.

In the 217 patients evaluated here, 31.8% were at standard risk and 34.1% were CD20 positive, which is in line with the frequencies in other studies [15, 16, 20, 21]. The proportion of CD20-positive patients in the standard-risk and high-risk groups was not significantly different [15, 16]. Thus, about 20% and 10% of the patients with Ph-negative BCP-ALL in our study were CD20-negative and CD20-positive standard-risk, respectively. Our analysis suggests a favorable prognosis for CD20-negative standard-risk BCP-ALL patients, despite being treated with chemotherapy alone. Within the standard-risk patients, we found that CD20-negative patients had a CR rate of 100% and better long-term outcomes compared with CD20-negative patients. With chemotherapy alone, the 3-year DFS and OS rates for these patients were 52.1% and 55.8%, respectively. However, the dissatisfactory response and survival rates of the CD20-positive standard-risk patients did not differ from those of high-risk patients treated without allo-HCT. This patient group may benefit from additional therapeutic modalities.

Allo-HCT is a standard treatment option for high-risk adult ALL patients [22, 23]. Our previous retrospective analysis showed superior survival of patients receiving haploidentical allo-HCT at CR1, with a 3-year DFS of ∼70%, independent of risk status [24, 25] Our preliminary analysis of 81 patients who received allo-HCT in CR1 in this cohort shows significant improved DFS and OS in all risk groups. We are working on the study to clarify the role of allo-HCT in patients with BCP-ALL. Patients with CD20-positive standard-risk ALL have a poor prognosis and are also supposed to benefit from allo-HCT. However, it should be noted that the lower rate of durable CR1 in patients with CD20-positive ALL may cause difficulties in bringing patients to allo-HCT in CR1.

Studies from the MD Anderson Cancer Center and the randomized GRAALL-R 2005 study have demonstrated that survival rates of patients with CD20-positive ALL are higher when treated with a combination of rituximab and chemotherapy compared with chemotherapy alone [26–28]. The addition of L-asparaginase may also benefit CD20-positive patients [29]. With a minimal residual disease-adapted therapeutic strategy, CD20-positive ALL patients did not have poorer outcome than CD20-negative patients as demonstrated by the NILG-ALL 09/00 protocol [20]. All these studies suggest therapeutic options to alleviate the negative effect of CD20 expression in patients with ALL.

The major limitation of our study is retrospective nature. Therefore, further studies to compare chemotherapy and allo-HCT in CR1 for CD20-negative standard-risk patients are warranted. Moreover, it will be interesting to investigate whether rituximab can significantly improve the prognosis of patients with CD20-positive standard-risk ALL. For the time being, our results suggest that CD20 expression adds to the known risk factors for Ph-negative BCP-ALL patients.

## MATERIALS AND METHODS

### Study design and the patients

**Figure 2:**
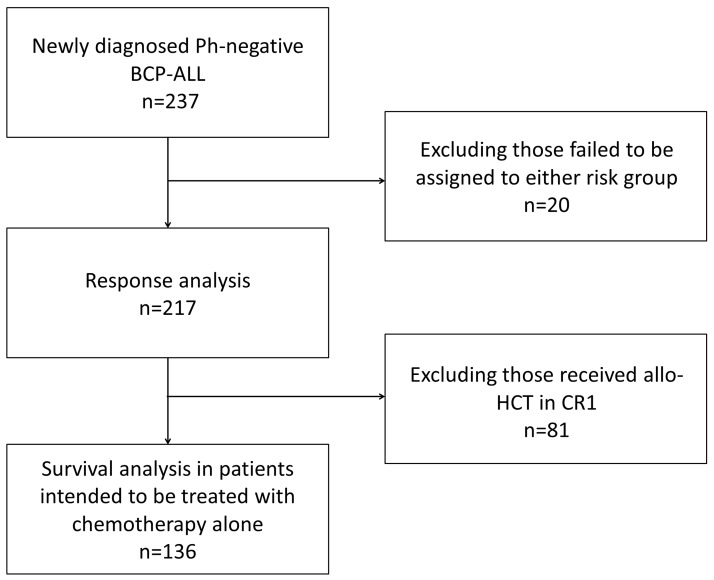
Study design: 237 consecutive patients aged ≥18 and <65 years with a diagnosis of de novo Ph-negative BCP-ALL were included in the study 217 patients could be assigned to standard-risk or high-risk groups were analyzed for responses to induction chemotherapies. 136 patients were intended to be treated with chemotherapy alone and were analyzed for long-term outcomes.

We conducted a retrospective study to evaluate the significance of ALL cell CD20 expression within the traditional risk stratifications and to redefine standard-risk BCP-ALL. We enrolled 237 consecutive patients aged ≥18 and <65 years with a diagnosis of de novo Ph-negative BCP-ALL who were treated at Peking University Institute of Hematology from January 2000 to February 2015. Twenty patients could not be assigned to standard-risk or high-risk groups because of indeterminate karyotype and absence of other high-risk factors, and they were excluded from the analysis. Data from the final cohort of 217 patients were analyzed for responses to induction chemotherapies. Eighty-one patients who achieved durable CR1 (≥3 months) received allogeneic transplantation according to their choice and donor availability. The remaining 136 patients were intended to be treated with chemotherapy alone and were analyzed for long-term outcome (Figure 2). All patients provided informed consent for treatment under a protocol reviewed and approved by Peking University Institute of Hematology. The last follow-up was conducted in October 2015.

### Diagnosis

Ph-negative BCP-ALL was diagnosed according to the WHO classification with morphologic, immunophenotypic, cytogenetic, and molecular analyses of bone marrow. Briefly, bone marrow smears were processed with Wright–Giemsa staining and viewed by experienced hematopathologists. We used 4-color and 8-color flow cytometry for leukemia diagnosis before and after 2009. PerCP-Cy5.5-conjugated anti-CD20 (Biolegend, San Diego, CA, USA) was used in 8-color flow cytometry and FITC conjugated anti-CD20 (BD, San Jose, CA, USA) in 4-color flow cytometry. CD20-positivity was defined as expression of CD20 on ≥20% of leukemia blasts. The positivity of CD20 expression determined by both 8-color and 4-color flow cytometry was comparable. Cytogenetic analysis was performed on bone marrow specimens after short-term (24 h) culture using the G-banding technique. Ph-negativity was confirmed by the absence of BCR-ABL fusion transcripts by TaqMan-based real-time (RT)-PCR and fluorescence *in situ* hybridization analysis. Expression of other leukemia-associated genes, such as MLL and E2A-PBX1, was also evaluated by TaqMan-based RT-PCR.

CNSL was diagnosed when the cerebrospinal fluid (CSF) leukocyte count was ≥5 × 10^6^/L or when blasts were detected in cytocentrifuged CSF specimens. EMD was defined as pathologic or radiologic evidence of disease in organs or tissue other than the blood or bone marrow (e.g., CNS, soft tissue, testes, skin, liver, or spleen).

### Risk stratification

Patients were evaluated according to the well-established risk factors of WBC count ≥30 × 10^9^/L and age ≥35 years old [30–32]. Patients were divided into four risk subgroups by cytogenetic and molecular abnormalities based on the MRC UKALLXII/ECOG E2993 adult ALL classification [5, 33] (Table 5). After modification according to the GRAALL trial [16], high-risk was defined as at least one of the following factors at baseline: age ≥35 years, WBC ≥30 × 10^9^/L, CNSL, and high- or very high-risk cytogenetic abnormality.

**Table 5:** The cytogenetic and molecular genetic prognostic risk stratificationfor adult Philadelphia chromosome-negative B cell precursor acute lymphoblastic leukemia [5, 33]

Standard risk	high hyperdiploidy with 51-65 chromosomes
Intermediate risk	normal, abnormalities of 11q (not MLL), del(6q), del(17p), del(12p), −13/del(13q), t(14q32), t(10;14), low hyperdiploidy (47-50), tetraploidy (>80, no structural changes), all others
High risk	−7, del(7p), +8, other 11q23/MLL translocations, t(1;19) or t(17;19)
Very high risk	t(4;11)/AF4/MLL+, t(8;14)/MYC/IGH+, complex karyotype (≥5 abnormalities) with or without translocations, combined low hypodiploidy (30-39)/near triploidy (60-78)

### Treatments

All patients were administered CODP±L as the induction regimen (cyclophosphamide 800 mg/m^2^ on day 1; vincristine 1.4 mg/m^2^ on days 1, 8, 15, and 22; daunorubicin 45 mg/m^2^ on days 1–3 and 15–17; prednisone 1 mg/kg on days 1–19 followed by tapering to cessation on day 28; with or without L-asparaginase 6000 U/m^2^ on days 19–28). Different consolidation regimens were used before and after 2010. After 2010, two regimens were used: alternative modified hyper-CVAD B (methotrexate 1 g/m^2^ on day 1 and cytarabine 1–2 g/m^2^ every 12 h on days 1–3) or hyper-CVAD A (cyclophosphamide 300 mg/m^2^ every 12 h on days 1–3; dexamethasone 40 mg/day on days 1–4 and 11–14; vincristine 1.4 mg/m^2^ on days 4 and 11; and doxorubicin 50 mg/m^2^ on day 4). Before 2010, three regimens were used: alternative CODP±L (cyclophosphamide 750 mg/m^2^ on day 1; vincristine 1.4 mg/m^2^ on day 1; daunorubicin 45 mg/m^2^ on day 1; prednisone 1 mg/kg on days 1–7; with or without l-asparaginase 6000 U/m^2^ on days 8–17), high dose methotrexate (1–1.5 g/m^2^ on day 1), or CAM (cyclophosphamide 800 mg/m^2^ on day 1; cytarabine 100/m^2^ on days 1–7; and mercaptopurine 75 mg/m^2^ on days 1–7). Patients received consolidation chemotherapy for 6 to 8 cycles. Subsequently, they received maintenance therapy for 2 years consisting of a combination of 6-mercaptopurine (75 mg/m^2^/day), methotrexate (20 mg/m^2^/week), vincristine (1.4 mg/m^2^/month, capped at 2 mg), and prednisone (60 mg/m^2^ on days 1–5 monthly). CNSL prophylaxis consisted of intrathecal chemotherapy with methotrexate 10 mg, cytarabine 50 mg, and dexamethasone 5 mg for at least 8 doses during induction and consolidation chemotherapy. Patients with active CNSL were administered intrathecal chemotherapy twice a week until the CSF examination was negative, followed by a regular schedule of intrathecal injections. EMD (except CNSL) was treated with systemic chemotherapy. Rituximab was not included in the protocol.

Consenting patients with appropriate donors received allo-HCT during CR1. The outcomes of those patients are not discussed here.

### Response criteria and the outcome assessment

CR was defined as (i) ≤5% blasts in normocellular marrow or hypercellular marrow with absolute neutrophil count ≥1 × 10^9^/L, (ii) platelets ≥100 × 10^9^/L, and (iii) resolution of EMD. CR duration ≥4 weeks was required as CR criterion. Relapse was defined as reappearance of (i) blasts in the blood, (ii) >5% blasts in bone marrow, or (iii) evidence of EMD following a CR. DFS was measured from CR until relapse or death. OS was measured from initiation of treatment until death.

### Statistical analysis

Statistical analyses were performed using SPSS version 17.0 (IBM-SPSS, Chicago, IL, USA). Comparison of patient characteristics based on CD20 expression was performed with chi-square test. Responses of standard-risk vs high-risk patients (total and CD20 stratified) were compared with chi-square test. Potential risk factors were evaluated by univariate and multivariate analysis. Potential risk factors with P values ≤0.2 in univariate analysis were entered into the multivariate analysis. DFS and OS were analyzed by Kaplan–Meier survival curves. Hazard ratios (HRs) for all variables were determined by Cox proportional hazard regression models. The Impact of risk factors on CIR was analyzed by a competing risk analysis using R project and the cmprsk package. All reported P values were two-sided. Statistical significance was set at P<0.05.
